# Inequalities involving hypergeometric and related functions

**DOI:** 10.1186/s13660-018-1842-4

**Published:** 2018-09-21

**Authors:** Siegfried H. Lehnigk

**Affiliations:** Leesburg, USA

**Keywords:** 33C05, 33B20, 26D10, 97K80, 65C60, Hypergeometric functions, Gamma functions, Inequalities, Applied statistics, Computational problems in statistics

## Abstract

An inequality is being proved which is connected to cost-effective numerical density estimation of the hyper-gamma probability distribution. The left-hand side of the inequality is a combination of two in the third parameter distinct versions of the hypergeometric function at the point one. All three parameters are functions of the distribution’s terminal shape. The first and second are equal. The distinct third parameters of the two hypergeometric functions depend on terminal and initial shape. The other side of the inequality is determined by the quotient of two infinite series, which are related to the first derivatives with respect to terminal shape of the hypergeometric functions which appear in its left-hand side.

## Introduction

Certain inequalities shall be considered, which involve combinations of gamma and psi functions of one positive variable *β* and one parameter *x* greater than unity. The basic functions involved are particular values of the hypergeometric function $F(a,b,c;s)$ [1, 9.122.1], namely:

1.1 $$\begin{aligned}& A(\beta; x) = F \biggl( \frac{1}{\beta }, - \frac{1}{\beta }, \frac{x}{\beta }; 1 \biggr) = \frac{\Gamma^{2} (x/\beta)}{\Gamma ( (x-1)/\beta ) \Gamma ( (x+1)/\beta ) } \\& \hphantom{ A(\beta; x)}= \prod_{\nu =0}^{\infty } \frac{(y_{\nu }- 1)(y_{\nu }+ 1)}{y_{\nu }^{2}} = \prod_{ \nu =0}^{\infty } \biggl( 1 - \frac{1}{y_{\nu }^{2}} \biggr), \end{aligned}$$

1.2 $$\begin{aligned}& B(\beta; x) = F \biggl( \frac{1}{\beta }, - \frac{1}{\beta }, \frac{x+1}{\beta }; 1 \biggr) = \frac{\Gamma^{2} ((x+1)/\beta)}{\Gamma ( x/\beta ) \Gamma ( (x+2)/\beta ) } \\& \hphantom{B(\beta; x) }= \prod_{\nu =0}^{\infty } \frac{y_{\nu }(y_{\nu }+ 2)}{(y_{\nu }+1)^{2}} = \prod_{\nu =0} ^{\infty } \biggl( 1 - \frac{1}{(y_{\nu }+ 1)^{2}} \biggr), \end{aligned}$$

where $\Gamma (s)$ is the gamma function, $y_{\nu }= \nu \beta + x$, $\nu = 0,1,2,\dots $, with $\beta \in (0, \infty)$, $x \in (1, \infty)$ throughout. The product representations of $A(\beta; x)$ and $B(\beta; x)$ are given is [1, 8.325.1]. Associated with these functions is their product

1.3 $$\begin{aligned} C(\beta; x) =& A(\beta; x) B(\beta; x) = F \biggl( \frac{1}{\beta }, - \frac{2}{\beta }; \frac{x}{\beta }; 1 \biggr) = \frac{\Gamma (x/\beta) \Gamma ((x+1)/\beta)}{\Gamma ( (x-1)/ \beta ) \Gamma ( (x+2)/\beta ) } \\ =& \prod_{\nu =0}^{\infty } \frac{(y_{\nu }- 1)(y_{\nu }+ 2)}{y_{\nu }(y_{\nu } +1)} = \prod_{\nu =0}^{\infty } \biggl( 1 - \frac{2}{y_{\nu }(y_{\nu } + 1)} \biggr). \end{aligned}$$

Since $a+b-c < 0$ for each of the hypergeometric functions in (1.1), (1.2), and (1.3), each of their series expansions converges throughout the entire closed unit circle of the complex plane [1, 9.102.2], and the gamma function expressions are justified [1, 9.122.1].

For the sake of simplicity we shall from now on omit the arguments *β* and *x* whenever there is no chance for confusion, keeping in mind, however, that *β* in the actual independent variable, and that *x* is a parameter. If we attach an argument to a symbol of a dependent variable, it will be a particular value of *β*. For example, $A(1) = A(1;x)$. Furthermore, derivatives will always be with respect to *β* and will be denoted by a prime.

The functions *A*, *B*, and *C* satisfy the inequalities

1.4 $$\begin{aligned}& 0 < C < A < B < 1. \end{aligned}$$

The infinite product representations of *A*, *B*, and *C* show that

1.5a $$\begin{aligned}& A \downarrow 0,\qquad B \downarrow 0,\qquad C \downarrow 0\quad \text{as } \beta \downarrow 0, \end{aligned}$$

and

1.5b $$\begin{aligned}& A \uparrow \frac{x^{2} - 1}{x^{2}} = A_{\infty },\qquad B \uparrow \frac{x(x +2)}{(x+1)^{2}} = B_{\infty },\qquad C \uparrow \frac{(x - 1((x+2)}{x(x+1)} = C_{\infty }, \end{aligned}$$

as $\beta \uparrow \infty $. (The down-arrow means that the function at its left decreases toward the value at its right as its argument decreases. The up-arrow indicates the opposite.) We note the particular values

1.6 $$\begin{aligned}& A(1) = \frac{x-1}{x},\qquad B(1) = \frac{x}{x+1},\qquad C(1) = \frac{x-1}{x+1}. \end{aligned}$$

The *β*-derivatives of *A*, *B*, and *C* are

1.7 $$\begin{aligned}& A' = 2AS,\qquad B' = 2BT, \qquad C' = 2C(S+T), \end{aligned}$$

with

1.8 $$\begin{aligned}& S(\beta; x) = \frac{1}{2\beta^{2}} \bigl[ (x-1) \psi_{1} - 2x \psi_{2} + (x+1) \psi _{3} \bigr] , \end{aligned}$$

1.9 $$\begin{aligned}& T(\beta; x) = \frac{1}{2\beta^{2}} \bigl[ x \psi_{2} - 2(x+1) \psi_{3} + (x+2) \psi _{4} \bigr] , \end{aligned}$$

$\psi_{\nu }= \psi ((x+\nu -2)/\beta)$, $\nu = 1, \dots,4, \psi (s)$ being the *ψ* function, $\psi (s) = d\log \Gamma (s)/ds$ [1, 8.360].

If we consider instead of (1.1), (1.2), and (1.3) the more general function

$$F(\varphi_{1}, \varphi_{2}, \varphi_{3};1) = \frac{\Gamma (\varphi_{3}) \Gamma (\varphi_{3} - \varphi_{1} - \varphi _{2})}{\Gamma (\varphi_{3} - \varphi_{1}) \Gamma (\varphi_{3} - \varphi_{2})} $$

with differentiable functions $\varphi_{\nu }= \varphi_{\nu }(\beta)$, $\nu = 1,2,3$, then

$$\begin{aligned} F' =& \bigl[ \varphi '_{3} \psi ( \varphi_{3}) + (\varphi_{3} - \varphi _{1} - \varphi_{2})' \psi (\varphi_{3} - \varphi_{1} - \varphi_{2}) \\ &{} - (\varphi_{3} - \varphi_{1})' \psi ( \varphi_{3} - \varphi _{1}) - (\varphi_{3} - \varphi_{2})' \psi (\varphi_{3} - \varphi_{2}) \bigr] F, \end{aligned}$$

provided that Re$(\varphi_{1} + \varphi_{2} - \varphi_{3}) < 0$ in some region *R* of the complex *β*-plane. For $\varphi_{1} = 1/\beta$, $\varphi_{2} = -1/\beta$, $\varphi_{3} = x/\beta $ this formula reduces to $A' = 2AS$ with *S* given in (1.8).

Related to *S* and *T* is the function

1.10a $$\begin{aligned} Q(\beta; x) =& \frac{1}{3} (S + 2T) \end{aligned}$$

1.10b $$\begin{aligned} =& \frac{1}{6\beta^{2}} \bigl[ (x-1) \psi_{1} - 3(x+1) \psi_{3} + 2(x+2) \psi_{4} \bigr] . \end{aligned}$$

The series expansion of the psi function [1, 8.362, 1],

$$\begin{aligned}& \psi (s) = -\gamma - \frac{1}{s} + s\sum_{\nu = 1}^{\infty } \frac{1}{\nu (s + \nu)}, \end{aligned}$$

where *γ* is Euler’s constant, leads to the series representations of *T*, *Q*, and *S*,

1.11 $$\begin{aligned}& T = \sum_{\nu = 1}^{\infty } \frac{\nu (y_{\nu }- 1)}{\rho_{\nu }}, \qquad Q = \sum_{\nu = 1} ^{\infty } \frac{\nu y_{\nu }}{\rho_{\nu }}, \qquad S = \sum_{\nu = 1}^{ \infty } \frac{\nu (y_{\nu }+ 2)}{\rho_{\nu }}, \end{aligned}$$

1.12 $$\begin{aligned}& \begin{aligned} &\rho_{\nu }(\beta; x) = (y_{\nu }-1) y_{\nu }(y_{\nu }+ 1) (y_{ \nu }+ 2), \quad y_{\nu }= \nu \beta + x > 1, \\ &\hphantom{\rho_{\nu }(\beta; x)} = (\nu \beta)^{4} + \alpha_{1} (\nu \beta)^{3} + \alpha_{2} (\nu \beta)^{2} + \alpha_{3} (\nu \beta) + \alpha_{4}, \quad \nu = 1, 2, \ldots, \\ &\alpha_{1} (x) = 2(2x + 1),\qquad \alpha_{2} (x) = 6x (x+1) - 1,\qquad \alpha_{3}(x) = 2\bigl(2x^{3} + 3x^{2} - x - 1\bigr), \\ &\alpha_{4} (x) = (x-1) x(x+1) (x+2) = x\bigl(x^{3} + 2x^{2} - x - 2\bigr),\quad x > 1. \end{aligned} \end{aligned}$$

(Cancellation of the respective factors $y_{\nu }+ \alpha$ ($\alpha = -1, 0, 1, 2$) in (1.11) would not bring any advantage. In fact, it would hamper comparison of equally numbered terms of these series and related expressions.)

Under the restrictions on *β* and *x*, each of the series in (1.11) is positive, and

1.13 $$\begin{aligned}& 0 < T < Q < S. \end{aligned}$$

Therefore, the derivatives $A'$, $B'$, and $C'$ in (1.7) are positive, i.e., the functions *A*, *B*, and *C* are strictly monotonically increasing for $\beta > 0$ and, by (1.5a), (1.5b), bounded. (This belatedly justifies the direction of the arrows in the limit relations (1.5a), (1.5b).)

We now introduce the gamma function combination

1.14 $$\begin{aligned}& \sigma (\beta; x) = \frac{A(1 - B)}{1-A},\quad \beta \in (0, \infty), \text{fixed } x \in (1, \infty), \end{aligned}$$

with *A* and *B* defined in (1.1) and (1.2), respectively. The main objective of this paper is to establish the inequality

1.15 $$\begin{aligned}& \sigma < Q/S,\quad \beta \in (0, \infty), \text{fixed } x \in (1, \infty). \end{aligned}$$

It is a crucial prerequisite to an efficient numerical solution routine of the four-parameter hyper-gamma density estimation problem for a given statistical data set (observations) [2, Chap. 9.3]. The hyper-gamma distribution has important applications in chemical, biological, and physical processes. The four parameters of the distribution are shift, scale, initial shape $x>1$ (in the statistical four-parameter case $x > 2$), and terminal shape $\beta > 0$. The statistical parameters *β* and *x* are to be determined from a set of two simultaneous equations [2, (9.33), (9.34)]. These are derived from the first four moments of the four-parameter hyper-gamma probability density function [2, (9.3.1)], which are defined by combinations of gamma functions as they appear in (1.1) and (1.2). A considerable computational cost advantage is achieved if it is known that for every fixed value of *x*, say, one of these equations has exactly one solution *β*. This will be the case if (1.15) holds. (See [2, Chaps. 9.2, 9.3], equation (9.3.34) and the discussion preceding (9.3.36).)

## Approximating sequences for $Q/S$ and $T/S$

The series (1.11) and their *β*-derivatives converge (absolutely and) uniformly as functions of *β* on every closed subinterval $[a, b] \subset (0, \infty)$ for any fixed $x > 1$. To show this, we look at *Q*, for example,

$$\begin{aligned}& Q = \sum_{\nu = 1}^{\infty }q_{\nu }(\beta; x),\qquad q_{ \nu }= \nu \bigl[ (y_{\nu }-1) (y_{\nu }+ 1) (y_{\nu }+ 2) \bigr] ^{-1},\qquad y_{\nu }= \nu \beta + x. \end{aligned}$$

(Here the common factor $y_{\nu }$ in numerator and denominator has been canceled.) We have $0 < q_{\nu }< 1 /\nu^{2} \beta^{3} < 1 / \nu^{2} a ^{3}$. Thus, by the Weierstraßcriterion,

$$0 < Q < \frac{1}{\beta^{3}} \sum_{\nu = 1}^{\infty } \frac{1}{\nu^{2}} = \frac{1}{a^{3}} \zeta (2), $$

where $\zeta (s)$ is Riemann’s zeta function [1, 9.522.1]. The terms of the *β*-derivative $Q'$ of *Q* are

2.1 $$\begin{aligned}& q'_{\nu }= -\nu^{2} ( \gamma_{1\nu } + \gamma_{2\nu } + \gamma_{3\nu }), \end{aligned}$$

with

$$\begin{aligned}& \gamma_{1\nu } = \bigl[ (y_{\nu }- 1)^{2} (y_{\nu }+ 1) (y_{\nu }+ 2) \bigr] ^{-1}, \\& \gamma_{2\nu } = \bigl[ (y_{\nu }- 1) (y_{\nu }+ 1)^{2} (y_{\nu }+ 2) \bigr] ^{-1}, \\& \gamma_{3\nu } = \bigl[ (y_{\nu }- 1) (y_{\nu }+ 1) (y_{\nu }+ 2)^{2} \bigr] ^{-1}. \end{aligned}$$

Now, $0 < \gamma_{\mu \nu } < 1 / \nu^{4} \beta^{4}$, $\mu = 1, 2, 3$, $\nu = 1, 2, \dots$, and, hence, $|q'_{\nu }| < 3 / a^{4} \nu^{2}$. Therefore, $|Q'| < 3 \zeta (2) / a^{4}$, i.e., the series $Q' = \sum q'_{\nu }$ is absolutely and uniformly convergent on $[a, b]$. Since $Q' < 0$, *Q* is strictly monotonically decreasing as *β* increases. The second derivative of *Q* is positive. This follows immediately from (2.1). Thus, with $Q'' > 0$, the function *Q* is concave from above, and $|Q'|$ is strictly monotonically decreasing. Corresponding facts are true for *T* and *S*. Note that *T*, *Q*, and $S \uparrow \infty $ (monotonically) as $\beta \downarrow 0$, and that *T*, *Q*, and $S \downarrow 0$ (monotonically) as $\beta \uparrow \infty $.

The following particular values of these functions for $\beta = 1$ are of interest:

2.2 $$\begin{aligned}& T(1) = \frac{1}{2(x+1)}, \qquad Q(1) = \frac{3x+1}{6x(x+1)}, \qquad S(1) = \frac{1}{2x}. \end{aligned}$$

They are obtained from (1.9), (1.10a), (1.10b), and (1.8), respectively, and from the functional relation [1, 8.365.3] of the psi function.

We now define the sequence $\{ F_{n} (\beta; x)\}$ with elements

2.3 $$\begin{aligned}& F_{n} = \frac{Q_{n} (\beta; x)}{S_{n} (\beta; x)},\quad n = 1, 2, \dots, \end{aligned}$$

where $Q_{n}$ and $S_{n}$ are the partial sums of *Q* and *S*,

2.4a $$\begin{aligned}& Q_{n} = \sum_{\nu = 1}^{n} \frac{\nu y_{\nu }}{\rho_{\nu }} = \frac{1}{\lambda_{n} (\beta; x)} \bigl[ x r_{n} (\beta; x) + \beta p_{n} (\beta; x) \bigr] , \end{aligned}$$

2.4b $$\begin{aligned}& S_{n} = \sum_{\nu = 1}^{n} \frac{\nu (y_{\nu }+ 2)}{\rho_{\nu }} = \frac{1}{\lambda_{n} (\beta; x)} \bigl[ (x+2) r_{n} (\beta; x) + \beta p_{n} (\beta; x) \bigr] , \end{aligned}$$

with

2.5 $$\begin{aligned}& \textstyle\begin{cases} r_{n} = \rho_{2} \cdots \rho_{n} + 2\rho_{1} \rho_{3} \cdots \rho_{n} + \cdots + n\rho_{1} \cdots \rho_{n-1}, \quad r_{1} \equiv 1, \\ p_{n} = \rho_{2} \cdots \rho_{n} + 2^{2} \rho_{1} \rho_{3} \cdots \rho _{n} + \cdots + n^{2} \rho_{1} \cdots \rho_{n-1}, \quad p_{1} \equiv 1, \\ \lambda_{n} = \rho_{1} \cdots \rho_{n}, \\ r_{n} < p_{n}, \qquad nr_{n} > p_{n}, \\ r_{n+1} = r_{n} \rho_{n} + (n+1) \lambda_{n},\qquad p_{n+1} = p_{n} \rho_{n} + (n+1)^{2} \lambda_{n}, \quad n= 1, 2, \dots. \end{cases}\displaystyle \end{aligned}$$

We observe that $r_{n}$ and $p_{n}$ (both polynomials in *β* of degree $4(n-1)$) can be expressed as

2.6 $$\begin{aligned}& r_{n} = \lambda_{n} \sum _{\nu = 1}^{n} u_{\nu }( \beta;x),\qquad p_{n} = \lambda_{n} \sum_{\nu =1}^{n} v_{ \nu }(\beta; x), \qquad u_{\nu }= \frac{\nu }{\rho_{\nu }},\qquad v_{\nu }= \frac{\nu^{2}}{\rho_{\nu }}, \end{aligned}$$

and that the positive series

2.7 $$\begin{aligned}& \sum_{\nu = 1}^{n} u_{\nu } \quad \mbox{and} \quad \sum_{\nu = 1}^{n} v_{\nu } \end{aligned}$$

converge uniformly (for any $x \in (1, \infty)$) on every interval $[a, b] \subset (0, \infty)$. This follows immediately by the Weierstraßcriterion since

2.8 $$\begin{aligned}& u_{\nu }= \frac{\nu }{\rho_{\nu }} < \frac{1}{a^{4} \nu^{3}}, \qquad v_{\nu }= \frac{\nu^{2}}{\rho_{\nu }} < \frac{1}{a^{4} \nu^{2}}, \quad \beta \in [a,b], \end{aligned}$$

so that

$$\sum_{\nu =1}^{\infty }u_{\nu }< \frac{1}{a^{4}} \zeta (3), \qquad \sum_{\nu =1}^{\infty }v_{\nu }< \frac{1}{a^{4}} \zeta (2),\quad \beta \in [a,b]. $$

Furthermore,

2.9 $$\begin{aligned}& u'_{\nu }= - \frac{\nu \rho '_{\nu }}{\rho_{\nu }^{2}}, \qquad v'_{\nu }= - \frac{\nu^{2} \rho '_{\nu }}{\rho_{\nu }^{2}}, \end{aligned}$$

so that

$$\bigl\vert u'_{\nu } \bigr\vert < \frac{4}{a^{5} \nu^{3}}, \qquad \bigl\vert v'_{\nu } \bigr\vert < \frac{4}{a^{5} \nu^{2}}, \quad \beta \in [a, b], $$

and, consequently,

$$\sum_{\nu = 1}^{\infty } \bigl\vert u'_{\nu } \bigr\vert < \frac{4}{a^{5}} \zeta (3), \qquad \sum_{\nu = 1}^{\infty } \bigl\vert v'_{\nu } \bigr\vert < \frac{4}{a^{5}} \zeta (2),\quad \beta \in [a, b]. $$

The series

2.10 $$\begin{aligned}& \sum_{\nu =1}^{\infty }u'_{\nu }< 0,\qquad \sum_{\nu =1}^{\infty }v'_{\nu }< 0, \end{aligned}$$

converge absolutely and uniformly on every interval $[a, b] \subset (0, \infty)$. Note that the series (2.7) are strictly monotonically decreasing functions of $\beta \in (0, \infty)$.

We return to the functions $F_{n}$ defined by (2.3) and show that

2.11 $$\begin{aligned}& F_{n} < \frac{Q}{S}, \quad n=1,2,\dots, \qquad Q = \sum_{\nu =1}^{\infty }q_{\nu }(\beta; x), \qquad S = \sum_{\nu =1}^{\infty }s_{\nu }( \beta; x), \end{aligned}$$

i.e., that

2.12 $$\begin{aligned}& QS_{n} - SQ_{n} > 0. \end{aligned}$$

By (1.11)

$$S = Q + 2 \sum_{\nu = 1}^{\infty }u_{\nu }, $$

$u_{\nu }$ given in (2.8), so that (2.12) may be replaced by

$$QS_{n} - \Biggl( Q + 2\sum_{\nu = 1}^{n} u_{\nu } \Biggr) Q_{n} = Q(S_{n} - Q_{n}) - 2Q_{n} \sum_{\nu =1}^{n} u_{\nu }> 0. $$

Here we replace $S_{n} - Q_{n}$ by $2 \sum_{\nu =1}^{n} u_{ \nu }$ and obtain, after dropping the common factor 2,

$$\begin{aligned} Q \sum_{\nu =1}^{n} u_{\nu }- Q_{n} \sum_{\nu =1}^{ \infty }u_{\nu } =& \Biggl( Q_{n} + \sum_{\nu =n+1}^{\infty }q _{\nu } \Biggr) \sum_{\nu =1}^{n} u_{\nu }- Q_{n} \Biggl( \sum_{\nu =1}^{n} u_{\nu }+ \sum_{\nu =n+1}^{\infty }u _{\nu } \Biggr) \\ =& \Biggl( \sum_{\nu =n+1}^{\infty }q_{\nu } \Biggr) \Biggl( \sum_{\nu =1}^{n} u_{\nu } \Biggr) - \Biggl( \sum_{\nu =1} ^{n} q_{\nu } \Biggr) \Biggl( \sum _{\nu =n+1}^{\infty }u_{\nu } \Biggr). \end{aligned}$$

Comparing equally numbered terms of the two infinite series, we get

2.13 $$\begin{aligned}& q_{n+k} \Biggl( \sum_{\nu =1}^{n} u_{\nu } \Biggr) - \Biggl( \sum_{\nu =1}^{n} q_{\nu } \Biggr) u_{n+k},\quad k = 1, 2, \dots , \end{aligned}$$

or, with $q_{n+k} = (n+k) y_{n+k} / \rho_{n+k}$ and $u_{n+k} = (n+k) / \rho_{n+k}$,

$$y_{n+k} \sum_{\nu = 1}^{n} \frac{\nu }{\rho_{\nu }} - \sum_{\nu = 1}^{n} \frac{\nu y_{\nu }}{\rho_{\nu }}. $$

This difference is positive since $y_{n+k} > y_{\nu }$ for $\nu = 1, \dots, n$. Therefore, (2.13) is correct, and, consequently, (2.11) holds. With this result, and

$$F_{1} = \frac{Q_{1}}{S_{1}} = \frac{x + \beta }{x + 2 + \beta } < \frac{Q}{S} < 1, $$

we see that

2.14 $$\begin{aligned}& \frac{Q}{S} \uparrow 1, \quad \text{as } \beta \uparrow \infty \end{aligned}$$

(the upward arrow will be justified in Sect. 3), and that

2.15 $$\begin{aligned}& \frac{Q}{S} \geq F_{n} (0) = \frac{x}{x+2} \quad \text{as } \beta \downarrow 0. \end{aligned}$$

Next, we show that

2.16 $$\begin{aligned}& F_{n+1} > F_{n},\quad n= 1, 2, \dots. \end{aligned}$$

By means of (2.4a), (2.4b), the rational functions $F_{n}$ defined in (2.3) can be written as

2.17 $$\begin{aligned}& F_{n} = \frac{xr_{n} + \beta p_{n}}{(x+2) r_{n} + \beta p_{n}},\quad n = 1, 2, \dots . \end{aligned}$$

With this expression for $F_{n}$, inequality (2.16) changes into $r_{n} p_{n+1} > r_{n+1} p_{n}$. The recurrence relations for $r_{n}$ and $p_{n}$ in (2.5) lead to $(n+1) r_{n} > p_{n}$, which is correct. Thus (2.16) holds.

The facts established so far show that $\{ F_{n} \}$ is a positive, increasing, bounded above sequence that converges for every $\beta \in (0, \infty)$ and any fixed $x \in (1, \infty)$ as $n \uparrow \infty $. We want to show now that it converges to $Q/S$ uniformly on every subinterval $[a, b]$ of $(0, \infty)$.

Observing (2.12), we have to show that for every $\varepsilon > 0$ there exists $n_{0} = n_{0} (\varepsilon)$ such that

2.18 $$\begin{aligned}& 0 < QS_{n} - SQ_{n} < \varepsilon\quad \text{for every } n \geq n_{0} \text{ on } [a, b] \subset (0, \infty). \end{aligned}$$

Now,

$$\begin{aligned} QS_{n} - SQ_{n} &= \Biggl( Q_{n} + \sum _{\nu = n+1}^{\infty }q_{\nu } \Biggr) S_{n} - \Biggl( S_{n} + \sum_{\nu = n+1}^{\infty }s_{\nu } \Biggr) Q_{n} \\ &= S_{n} \sum_{\nu = n+1}^{\infty }q_{\nu }- Q_{n} \sum_{\nu = n+1}^{\infty }s_{\nu }. \end{aligned}$$

Since $q_{\nu }< s_{\nu }= \nu (y_{\nu }+ 2) / \rho_{\nu }$, we have

2.19 $$\begin{aligned}& QS_{n} - SQ_{n} < (S_{n} - Q_{n}) \sum_{\nu = n+1} ^{\infty }s_{\nu }. \end{aligned}$$

Here

$$S_{n} - Q_{n} = 2 \sum_{\nu =1}^{n} \frac{\nu }{\rho_{\nu }} < 2 \sum_{\nu =1}^{\infty } \frac{\nu }{\rho_{\nu }} = 2 \sum_{\nu =1}^{\infty }u_{\nu }. $$

The right-hand side of this inequality is strictly monotonically decreasing as stated earlier in connection with (2.8) and (2.10). Thus

$$\sum_{\nu =1}^{\infty } \frac{\nu }{\rho_{\nu }} < \sum _{\nu =1}^{\infty } \frac{\nu }{\rho_{\nu }(a)} = \frac{1}{2} K, \quad \beta \in [a, b]. $$

Therefore, we may continue inequality (2.19) as follows:

$$QS_{n} - SQ_{n} < K \sum_{\nu =1}^{\infty }s_{\nu }. $$

Uniform convergence of *S* on $[a, b]$ implies that, given $\varepsilon /K$, there exists $n_{0} = n_{0}(\varepsilon)$ such that

$$QS_{n} - SQ_{n} < K \frac{\varepsilon }{K} = \varepsilon \quad \text{on } [a,b] \text{ if } n \geq n_{0}. $$

This proves (2.18). So $\{ F_{n} \}$ is a uniformly convergent approximating sequence for $Q/S$ from below, and

$$\frac{Q}{S} = F_{1} + \sum_{\nu =1}^{\infty }(F_{\nu }- F_{\nu -1}). $$

This shows that $(Q/S) (0) = F_{1}(0) = x/(x+2)$, so that (2.15) may be sharpened to

2.20 $$\begin{aligned}& \frac{Q}{S} \downarrow F_{n} (0) = \frac{x}{x+2} \quad \text{as } \beta \downarrow 0. \end{aligned}$$

(The downward arrow will be justified in Sect. 3.)

Along the same lines analogous results can be established for the function $T/S$ and its approximating from below sequence

$$G_{n} = \frac{T_{n}}{S_{n}} = \frac{(x-1) r_{n} + \beta p_{n}}{(x+2) r_{n} + \beta p_{n}},\quad n = 1,2,\dots . $$

Note that

2.21 $$\begin{aligned}& \frac{T}{S} \downarrow G_{n} (0) = \frac{x-1}{x+2} \quad \text{as } \beta \downarrow 0, n = 1, 2, \dots, \end{aligned}$$

and

2.22 $$\begin{aligned}& \frac{T}{S} \uparrow 1 \quad \text{as } \beta \uparrow \infty . \end{aligned}$$

## Monotonicity of $Q/S$ and $T/S$

The functions $Q/S$ and $T/S$ are strictly monotonically increasing for $\beta \in (0, \infty)$. It is sufficient to show this for $Q/S$ since, by (1.10a), $T/S = (3Q/S-1)/2$.

We introduce the rational functions

3.1 $$\begin{aligned}& f_{n} (\beta; x) = \frac{r_{n}}{p_{n}},\quad n=1,2, \dots, \qquad f_{1} \equiv 1, \end{aligned}$$

$r_{n}$ and $p_{n}$ defined by (2.6). Note that $f_{n+1} < f_{n}$ because $\{ u_{\nu }/ v_{\nu }\} = \{ 1 / \nu \}$ is a decreasing sequence [3, p. 10, Problem 28]. The approximating functions $F_{n}$ in (2.17) can now be written as

3.2 $$\begin{aligned}& F_{n} = \frac{xf_{n} + \beta }{(x+2) f_{n} + \beta },\quad n=1,2,\dots, \qquad F_{n}(0) = \frac{x}{x+2}. \end{aligned}$$

The *β*-derivative of $F_{n}$ is

3.3 $$\begin{aligned}& F'_{n} = \frac{2}{[(x+2)f_{n} + \beta ]^{2}} \bigl(f_{n} - \beta f'_{n}\bigr), \quad n=1,2, \dots , \end{aligned}$$

and

$$F'_{n}(0) = \frac{2(2n+1)}{3(x+2)^{2}} > 0, \quad n=1,2,\dots . $$

This immediately shows that $F'_{n} > 0$ for small positive values of *β*. We want to show that $F'_{n} > 0$ for $\beta \in (0, \infty)$. But first we must establish certain facts about the rational functions $f_{n} = r_{n} / p_{n}$. By (2.5) and with $\rho_{\nu }= ( \nu \beta + x - 1)(\nu \beta + x)(\nu \beta + x + 1) (\nu \beta + x +2)$ the constituent terms of $r_{n}$ and $p_{n}$ are products of $n-1$ Hurwitz polynomials, each of degree 4, so that each of those terms is a Huwitz polynomial of degree $4(n-1)$. The *β*-derivative of $f_{n}$ is

3.4a $$\begin{aligned} f'_{n} =& \frac{1}{p_{n}^{2}} \bigl( r'_{n} p_{n} - r_{n} p'_{n} \bigr) \end{aligned}$$

3.4b $$\begin{aligned} =& \Biggl[ \Biggl( \sum_{\nu =1}^{n} u'_{\nu } \Biggr) \Biggl( \sum_{\nu =1}^{n} v_{\nu } \Biggr) - \Biggl( \sum_{\nu =1} ^{n} u_{\nu } \Biggr) \Biggl( \sum _{\nu =1}^{n} v'_{\nu } \Biggr) \Biggr] \Biggl( \sum_{\nu =1}^{n} v_{\nu } \Biggr) ^{-2}. \end{aligned}$$

Using (2.8) and (2.9) in (3.4b), we arrive at

3.5 $$\begin{aligned}& \Biggl( \sum_{\nu =1}^{n} v_{\nu } \Biggr) ^{2} f'_{n} = \sum _{1 \leq \mu < \nu \leq n}^{n} \frac{\mu \nu (\nu - \mu)}{\rho_{\mu }^{2} \rho_{\nu }^{2}} \bigl( \rho_{\mu }\rho '_{\nu }- \rho '_{\mu } \rho_{\nu } \bigr) . \end{aligned}$$

Here

$$\begin{aligned} \rho_{\mu }\rho '_{\nu }- \rho '_{\mu } \rho_{\nu }= {}& (\nu - \mu) \bigl\{ \mu^{3} \nu^{3} \alpha_{1} \beta^{6} + 2\mu^{2} \nu^{2} (\mu + \nu) \alpha_{2} \beta^{5} \\ &{}+ \bigl[ 3\mu \nu \bigl(\mu^{2} + \mu \nu + \nu^{2}\bigr) \alpha_{3} + \mu^{2} \nu ^{2} \alpha_{1} \alpha_{2} \bigr] \beta^{4} \\ &{}+ \bigl[ 4\bigl(\mu^{2} + \nu^{2}\bigr) (\mu + \nu) \alpha_{4} + 2\mu \nu ( \mu + \nu) \alpha_{1} \alpha_{3} \bigr] \beta^{3} \\ &{}+ \bigl[ 3\bigl(\mu^{2} + \mu \nu + \nu^{2}\bigr) \alpha_{1} \alpha_{4} + \mu \nu \alpha_{2} \alpha_{3}\bigr] \beta^{2} \\ &{}+ 2(\mu + \nu) \alpha_{2} \alpha_{4} \beta + \alpha_{3} \alpha _{4} \bigr\} > 0, \quad \beta \in (0, \infty), x \in (1, \infty), 1 \leq \mu < \nu \leq n, \end{aligned}$$

the positive functions $\alpha_{\nu }$ ($\nu = 1,\dots, 4$) being defined in connection with (1.12). Thus, by (3.5), $f'_{n} > 0$, $\beta > 0$, $x>1$. This establishes strict monotonicity of the functions $f_{n}$ defined in (3.1). Note that $0 < f_{n} < 1$, $n \geq 2$, $\beta > 0$ and

3.6 $$\begin{aligned}& \begin{aligned} & f_{n} \downarrow \frac{3}{2n+1} \quad \text{as } \beta \downarrow 0, \qquad f_{n} \uparrow \frac{1 + 1/2^{3} + \cdots + 1/n^{3}}{1 + 1/2^{2} + \cdots + 1/n^{2}} = \frac{d_{n}}{e_{n}} \quad \text{as } \beta \uparrow \infty , \\ &f_{n} (\infty) = \lim_{\beta \uparrow \infty } f_{n} \downarrow \zeta (3) / \zeta (2) = 0.730 763\dots \quad \text{as } n \uparrow \infty . \end{aligned} \end{aligned}$$

We also note the following facts about $f'_{n}$, which follow from (3.4a),

3.7 $$\begin{aligned}& f'_{n} \uparrow \frac{9\alpha_{3} (n^{2} + n -2)}{\alpha_{4} (2n+1)^{2}} > 0 \quad \text{as } \beta \downarrow 0,\qquad f'_{n} \downarrow 0\quad \text{as } \beta \uparrow \infty . \end{aligned}$$

Furthermore, (2.5) together with (1.12) and with the notation used in (3.6) shows that

$$\begin{aligned}& r_{n} = (n!)^{4} d_{n} \beta^{4(n-1)} + \text{lower order terms}, \\& p_{n} = (n!)^{4} e_{n} \beta^{4(n-1)} + \text{lower order terms}, \end{aligned}$$

so that

$$\begin{aligned}& r'_{n} = 4(n-1) (n!)^{4} d_{n} \beta^{(4(n-1)-1} + \cdots, \\& p'_{n} = 4(n-1) (n!)^{4} e_{n} \beta^{(4(n-1)-1} + \cdots . \end{aligned}$$

Consequently,

$$r'_{n} p_{n} - r_{n} p'_{n} = c_{n} \beta^{8(n-1)-2} + \text{lower order terms}, $$

where $c_{n}$ is a positive constant. Since

$$p_{n}^{2} = (n!)^{8} e_{n}^{2} \beta^{8(n-1)} + \text{lower order terms}, $$

we see that $f'_{n}$ as given in (3.4a) behaves like $\beta^{-2}$ as $\beta \uparrow \infty $. In other words, $f'_{n}$ and $\beta^{-2}$ are asymptotically proportional. Consequently,

3.8 $$\begin{aligned}& \beta f'_{n} \downarrow 0 \quad \text{as } \beta \uparrow \infty . \end{aligned}$$

We now remember the fact that the polynomials $\rho_{\nu }$ are Hurwitzian and that, consequently, the constituent terms of the polynomials $r_{n}$ and $p_{n}$ in (2.5) are Hurwitzian. By Theorem IV of [4] (in conjunction with the specification of terminology concerning *circular regions* and *circles* on p. 164 of [4]) the sum of any two of these constituent polynomials of degree $4(n-1)$ is Hurwitzian. Thus, $r_{n}$ and $p_{n}$ are Hurwitz polynomials. Their zeros are located in the open left-hand half of the complex *β*-plane, which we denote by *L*. By another theorem [5, p. 115], all zeros of $f'_{n}$ (and all its poles) are located in *L*. In other words, $f'_{n} > 0$ for real $\beta > 0$, a fact which has been established earlier already by direct means. Applying the theorem of [5] again, this time to the rational function $f'_{n}$, we arrive at the result that $f''_{n}$ has all its zeros (and poles) in *L*, i.e., $f''_{n} \neq 0$ for real $\beta > 0$. Limit relation (3.7) shows that $f'_{n}$ decreases somewhere in the interval $(0, \infty)$. Thus, the *β*-derivative $f''_{n}$ of $f'_{n}$ must be negative somewhere. Since $f''_{n} \neq 0$ for $\beta > 0$, if follows that $f''_{n} < 0$ for all $\beta > 0$. In other words, $f_{n}$, defined by (3.1) is concave from below on $0 < \beta < \infty $, i.e., the tangent at any point $(\beta_{0}, f_{n}(\beta_{0}))$, $\beta_{0} > 0$, lies above the graph of $f_{n}$ for every $\beta > 0$, $\beta \neq \beta_{0}$.

Since, by (3.2), $F'_{n} (0) > 0$, if follows that $F' > 0$ at least for small positive values of *β*. This means that $f_{n} - \beta f'_{n} > 0$ for small positive values of *β* as can be seen from (3.3). This can also be verified by means of (3.6), (3.7), and (3.8), respectively. By (3.6) and (3.8) we see that $f_{n} - \beta f'_{n} > 0$ also for large values of *β*, so that $F'_{n} > 0$ for large *β*. Suppose now that $f_{n} - \beta f'_{n} < 0$ at some point $\beta > 0$. Then there exist points $\beta_{1}$ and $\beta_{2}$, $0 < \beta_{1} < \beta_{2}$, such that

3.9 $$\begin{aligned}& f_{n} (\beta_{\nu }) = \beta_{\nu }f'_{n} (\beta_{ \nu }), \quad \nu = 1, 2, \end{aligned}$$

$f_{n} - \beta f'_{n} > 0$ for $0 < \beta < \beta_{1}$ and $\beta_{2} < \beta < \infty $, and $f_{n} - \beta f'_{n} < 0$ for $\beta_{1} < \beta < \beta_{2}$. Consequently, there would exist a continuous function $\alpha (\beta)$ such that $\alpha (\beta_{\nu }) = 0$, $\nu = 1, 2$, $\alpha (\beta) > 0$, $\beta_{1} < \beta < \beta_{2}$, and

$$f'_{n} - \frac{1}{\beta } f_{n} - \alpha = 0, \quad \beta_{1} < \beta < \beta _{2}. $$

This inhomogeneous linear differential equation has the unique solution

3.10 $$\begin{aligned}& f_{n} = \biggl( \frac{f_{n} (\beta_{1})}{\beta_{1}} \biggr) \beta + \beta \int _{\beta_{1}}^{\beta } \frac{\alpha (\tau)}{\tau } \,d\tau, \quad \beta_{1} \le \beta \le \beta_{2}, \end{aligned}$$

with initial condition $(\beta_{1}, f_{n} (\beta_{1}))$. Its derivative is

$$f'_{n} = \frac{f_{n} (\beta_{1})}{\beta_{1}} + \int _{\beta_{1}}^{\beta } \frac{\alpha (\tau)}{\tau } \,d\tau + \alpha ( \beta), \quad \beta_{1} \le \beta \le \beta_{2}. $$

Observing (3.9) for $\nu =2$ and noting that $\alpha (\beta_{2}) =0$, we have at $\beta_{2}$

3.11 $$\begin{aligned}& f'_{n} (\beta_{2}) = \frac{f_{n} (\beta_{1})}{\beta_{1}} + \int _{\beta_{1}}^{\beta _{2}} \frac{\alpha (\tau)}{\tau } \,d\tau = \frac{f_{n} (\beta_{2})}{\beta_{2}} > \frac{f_{n} (\beta_{1})}{\beta_{1}}, \end{aligned}$$

since the integral is positive. Now, the tangent to the integral curve defined by (3.10) at the point $(\beta_{1}, f_{n} (\beta_{1}))$ is given by $y(\beta) = f_{n} (\beta_{1}) + (\beta - \beta_{1}) f'_{n} (\beta _{1})$ with $f'_{n} (\beta_{1}) = f_{n}(\beta_{1}) / \beta_{1}$ by (3.9).

Thus,

$$y(\beta_{2}) = f_{n} (\beta_{1}) + ( \beta_{2} - \beta_{1}) \frac{f_{n} (\beta_{1})}{\beta_{1}} = \frac{f_{n} (\beta_{1})}{\beta_{1}} \beta_{2}. $$

Since $f_{\nu }$ is concave from below it follows that

$$y(\beta_{2}) = \frac{f_{n} (\beta_{1})}{\beta_{1}} \beta_{2} > f_{n} (\beta_{2}), $$

or

$$\frac{f_{n} (\beta_{2})}{\beta_{2}} < \frac{f_{n} (\beta_{1})}{\beta_{1}} $$

in contradiction to (3.11). Consequently, $f_{n} - \beta f'_{n} > 0$ for all $\beta \in (0, \infty)$, and, hence, as (2.3) shows, $F'_{n} > 0$ for $0 < \beta < \infty $, which means that $F_{n} = Q_{n} / S_{n}$, or $F_{n} = (xf_{n} + \beta) / [ (x+2)f_{n} + \beta ]^{-1}$, is a strictly monotonically increasing function of $\beta \in (0, \infty)$. A corresponding result holds for $G_{n} = T_{n} / S_{n}$.

We now show that the sequence $\{ F'_{n} \}$ converges uniformly on every closed interval $[a, b] \subset (0, \infty)$. Differentiating $F_{n} = Q_{n} / S_{n}$, we form

3.12 $$\begin{aligned}& \bigl\vert F'_{n+k} - F'_{n} \bigr\vert = \bigl(S_{n}^{2} S_{n+k}^{2}\bigr)^{-1} \bigl\vert S _{n}^{2} \bigl(Q'_{n+k} S_{n+k} - Q_{n+k} S'_{n+k}\bigr) - S_{n+k}^{2} \bigl(Q'_{n} S _{n} - Q_{n} S'_{n}\bigr) \bigr\vert . \end{aligned}$$

With $Q = \sum q_{\nu }$, $S = \sum s_{\nu }$, equation (3.12), after some manipulations, can be brought into the form

3.13 $$\begin{aligned}& \textstyle\begin{cases} \vert F'_{n+k} - F'_{n} \vert \\ \quad = ( S_{n}^{2} S_{n+k}^{2} ) ^{-1} \vert ( \sum_{\nu = n+1}^{n+k} s_{\nu } ) ( -S _{n}^{2} Q'_{n} + 2Q_{n} S_{n} S'_{n} + S_{n}^{2} ( \sum_{\nu = n+1}^{n+k} q'_{\nu } ) ) \\ \qquad {} + ( \sum_{\nu = n+1}^{n+k} s_{\nu } ) ^{2} ( Q'_{n} S_{n} - Q_{n} S'_{n} ) \\ \qquad {} - ( \sum_{\nu = n+1}^{n+k} s'_{ \nu } ) ( S_{n}^{2} Q_{n} + S_{n}^{2} ( \sum_{\nu = n+1}^{n+k} q_{\nu } ) ) \\ \qquad {} - ( \sum_{\nu = n+1}^{n+k} q_{\nu } ) S_{n} ^{2} S'_{n} + ( \sum_{\nu = n+1}^{n+k} q'_{\nu } ) S _{n}^{3} \vert . \end{cases}\displaystyle \end{aligned}$$

Here, we replace all negative terms between the absolute value bars by their absolute values. Since *Q*, $|Q'|$, *S*, $|S'|$ are series with positive terms, we increase the right-hand side of (3.13) by replacing their partial sums by the entire series. Then we remember the fact that these series are monotonically decreasing functions of *β*. Therefore, we increase the right-hand side of (3.13) further by setting $Q_{n} < Q < Q(a)$, $|Q'_{n}| < |Q'| < |Q'(a)|$, $S_{n} < S(a)$, $|S'_{n}| < |S'(a)|$ for $\beta \in [a,b]$. We may also replace $\sum q_{\nu }$ by $\sum s_{\nu }$ since $0 < q_{\nu }< s_{\nu }$. Thus, there exist positive constants $K_{\nu }$ ($\nu = 1,\dots,5$) such that equality (3.13) may be replaced by the inequality

3.14 $$\begin{aligned}& \textstyle\begin{cases} \vert F'_{n+k} - F'_{n} \vert < ( S_{n}^{2} S_{n+k}^{2} ) ^{-1} \{ K_{1} \sum_{\nu = n+1}^{n+k} s_{\nu }+ K_{2} ( \sum_{\nu = n+1}^{n+k} s_{\nu } ) ^{2} + K_{3} \sum_{\nu = n+1}^{n+k} \vert s'_{\nu } \vert \\ \hphantom{ \vert F'_{n+k} - F'_{n} \vert < } {} + K_{4} \sum_{\nu = n+1}^{n+k} s_{\nu }+ K_{5} \sum_{\nu = n+1}^{n+k} \vert q'_{\nu } \vert \} . \end{cases}\displaystyle \end{aligned}$$

Furthermore, by (1.11),

$$\begin{aligned} S_{n+k} >{} & S_{n} > S_{1} = \bigl[ (\beta + x -1) (\beta + x) (\beta + x +1) \bigr] ^{-1} \\ >{} & \bigl[ (b+x-1) (b+x) (b+x+1) \bigr] ^{-1} = B^{-1} > 0, \quad \beta \in [a,b], \end{aligned}$$

so that

3.15 $$\begin{aligned}& \bigl( S_{n}^{2} S_{n+k}^{2} \bigr) ^{-1} < \bigl( S _{n}^{4} \bigr) ^{-1} < \bigl( S_{1}^{4} \bigr) ^{-1} < B^{4},\quad \beta \in [a, b]. \end{aligned}$$

Now, since *S*, $S'$, and $Q'$ converge uniformly on $[a, b] \in (0, \infty)$, given $\varepsilon > 0$ there exists a number $n_{0}( \varepsilon)$ such that each of the five finite sums in (3.14) is less than $B^{-4} (\sum_{\nu =1}^{5} K_{\nu })^{-1} \varepsilon $ for every $n \geq n_{0}$ and for all $k \geq 1$. Thus (3.14) together with (3.15) leads to $|F'_{n+k} - F'_{n}| < \varepsilon $ for every $n \geq n_{0}$ and for all $k \geq 1$ on every $[a, b] \subset (0, \infty)$. The final result is that $\{ F'_{n} \}$ converges uniformly for $\beta \in [a,b]$ to the function $(Q/S)' > 0$, and this means that $Q/S$ is strictly monotonically increasing. (This result justified the direction of the arrows in (2.14) and (2.20), and in (2.21) and (2.22) for $T/S$.)

## Properties of *σ*

The function

4.1 $$\begin{aligned}& \sigma = \frac{A(1-B)}{1-A}, \quad \beta \in (0, \infty), \text{fixed } x \in (1, \infty), \end{aligned}$$

together with (1.1) and (1.2), evidently satisfies the inequality $0 < \sigma < A$, $\beta > 0$ and by (1.5a), (1.5b), the limit relations

4.2 $$\begin{aligned}& \sigma \downarrow 0 \quad \text{as } \beta \downarrow 0, \qquad \sigma \downarrow \frac{x-1}{x+1} = \sigma_{\infty }\quad \text{as } \beta \uparrow \infty . \end{aligned}$$

(The downward arrows in (4.2) will be justified momentarily.) Furthermore, observing (1.6), we see that at $\beta = 1$

4.3 $$\begin{aligned}& \sigma (1) = \sigma_{\infty }= C(1) = \frac{x-1}{x+1}. \end{aligned}$$

The *β*-derivative of *σ* is given by

4.4 $$\begin{aligned}& \sigma ' = \frac{2S}{1-A} \biggl( \sigma - C \frac{T}{S} \biggr) . \end{aligned}$$

This shows that $\sigma < Q/S$ whenever $\sigma '$ is nonpositive. For, if $\sigma ' \le 0$, then $\sigma \le CT/S < T/S < Q/S$, if we observe (1.3) and (1.4). The function $\sigma '$ is negative somewhere. To see this, we note that for $\beta = 1$, (4.4) becomes

4.5 $$\begin{aligned}& \sigma ' (1) = \frac{x-1}{4(x+1)^{2}} > 0. \end{aligned}$$

Here (1.6) and (2.2) have been used. Continuity of *σ* implies that $\sigma (1 + \varepsilon) > \sigma (1) = \sigma_{\infty }$ at least for sufficiently small values of $\varepsilon > 0$. Inequality (4.5) and the second limit relation (4.2) show that *σ* has a local maximum at some point $\beta^{\star }> 1$. Suppose *σ* had more than one maximum in $(0, \infty)$. Let $0 < \beta_{1} < \beta _{2}$ be points at which maxima are attained. Then *σ* must have a minimum at some point $\beta_{0}$, $\beta_{1} < \beta_{0} < \beta _{2}$. We have

4.6 $$\begin{aligned}& \sigma (\beta_{1}) > \sigma (\beta_{0}), \end{aligned}$$

and $\sigma ' (\beta_{1}) = \sigma ' (\beta_{0}) = 0$, so that, according to (4.4)

$$\sigma (\beta_{1}) = C(\beta_{1}) \frac{T(\beta_{1})}{S(\beta_{1})}, \qquad \sigma (\beta_{0}) = C(\beta _{0}) \frac{T(\beta_{0})}{S(\beta_{0})}. $$

Since *C* and $T/S$ are both monotonically increasing, it would follow that $\sigma (\beta_{1}) < \sigma (\beta_{0})$, in contradiction to (4.6). Consequently, there exists one and only one point $\beta^{ \star }$ at which $\sigma ' = 0$ and at which *σ* takes its maximum. We have $\sigma ' \geq 0$ if $\beta \in (0, \beta^{\star }]$, $\sigma ' < 0$ if $\beta \in (\beta^{\star }, \infty)$. In other words, *σ* is strictly monotonically decreasing on $(\beta^{\star }, \infty)$. (This result belatedly justifies the direction of the arrows in the limit relations (4.2).)

We now turn to the relation between *σ* given by (4.1) and $C = AB$ defined by (1.3). By (4.3), $\sigma (1) = C(1)$. Furthermore, by (4.2) and (1.5b) $\sigma_{\infty }< C_{\infty }$. Thus, *σ* and *C* are not identical, and $\sigma < C$ for large values of *β* since *σ* is strictly monotonically decreasing after it reaches its single maximum at $\beta^{\star }> 1$, and *C* is strictly monotonically increasing. More can be obtained by comparing the derivatives of *σ* and *C* at $\beta = 1$. Using the particular value of *C* at $\beta = 1$ given in (1.6) and those of *S* and *T* for $\beta = 1$ given in (2.2) and the definition of $C'$ given in (1.7), we find that

$$C'(1) = \frac{(x-1)(2x+1)}{x(x+1)^{2}}. $$

Comparing this with $\sigma ' (1)$ in (4.5), we see that

$$C'(1) = 4 \frac{2x+1}{x} \sigma '(1) > \sigma '(1). $$

This and $\sigma (1) = C(1)$ imply that for sufficiently small $\varepsilon > 0$,

4.7 $$\begin{aligned}& \sigma < C, \quad \beta \in (1, 1+\varepsilon), \end{aligned}$$

and

4.8 $$\begin{aligned}& \sigma > C, \quad \beta \in (1-\varepsilon, 1). \end{aligned}$$

We show first that (4.7) holds for $\beta \in (1, \infty)$. Observing (4.1) and setting $C = AB$, we transform the desired inequality (4.7) into

4.9 $$\begin{aligned}& AB - 2B+1 < 0. \end{aligned}$$

Here, we set $A = \kappa B$, $0 < \kappa = \kappa (\beta; x) < 1$, $\beta \in [1, \infty)$, $x \in (1, \infty)$, noting that *κ* has a positive *β*-derivative (by (1.7) and (1.13)),

$$\kappa ' = 2\kappa (S-T) > 0, $$

so that *κ* is strictly monotonically increasing, and by (1.6) and (1.5b),

$$\kappa (1) = \frac{x^{2} - 1}{x^{2}}, \qquad \kappa_{\infty }= \lim _{\beta \uparrow \infty } \kappa = \frac{A_{\infty }}{B_{\infty }} = \frac{(x^{2} - 1)(x+1)^{2}}{x^{3} (x+2)}. $$

Furthermore, since by (1.1) and (1.2), the factors of the infinite product for $\kappa = A/B$ go to $(x+1)^{3} (x-1) / x^{3} (x+2) < 1$ as $\beta \downarrow 0$, *κ* diverges toward 0 as $\beta \downarrow 0$.

With $A = \kappa B$, inequality (4.9) takes the form

4.10 $$\begin{aligned}& g(B) = B^{2} - \frac{2}{\kappa } B + \frac{1}{\kappa } < 0. \end{aligned}$$

The equation $g(B) = 0$ has the two roots,

$$\begin{aligned}& B_{1} = \frac{1}{\kappa } (1 - \sqrt{1-\kappa }),\qquad 0 < \kappa = AB^{-1} < 1, \qquad B_{1} \bigl(\kappa (1)\bigr) = B(1), \\& B_{2} = \frac{1}{\kappa } (1 + \sqrt{1-\kappa }), \end{aligned}$$

$0 < B_{1} < 1 < B_{2}$. Since $B < B_{\infty }= x(x+2) / (x+1)^{2} < B_{2}$ by (1.5b), the root $B_{2}$ is outside the range of *B*. At $B_{\infty }$, *g* takes the negative value $g(B_{\infty }) = -2 [x(x+1)]^{-1}$. Consequently, as stipulated by (4.10), $g(B) < 0$ for $B_{1} < B < B_{\infty }$, i.e., for $1 < \beta < \infty $, and $g(B) = 0$ if and only if $\beta = 1$, i.e., for $B(1) = B_{1} ( \kappa (1))$. Thus (4.7) holds for $\beta \in (1, \infty)$.

For $\beta \in (0,1)$ we have $0 < B < B(1) = B_{1} (\kappa (1))$, and the function $g(B)$ in (4.10) is positive, i.e., the inequality sign in (4.10) is reversed. This proves (4.8) for the entire interval $0 < \beta < 1$.

Our results are these:

$$\begin{aligned}& C < \sigma, \quad \beta \in (0,1), \text{fixed } x \in (1, \infty), \\& \sigma < C,\quad \beta \in (1,\infty), \text{ fixed } x \in (1, \infty), \end{aligned}$$

and

$$\begin{aligned}& \sigma = C \quad \text{if and only if}\quad \beta = 1, \text{fixed } x \in (1, \infty). \end{aligned}$$

Finally, for $\beta = 1$, $g(B(1)) = 0$, i.e.,

4.11 $$\begin{aligned}& C - 2B + 1 = 0,\quad \text{if and only if}\quad \beta = 1. \end{aligned}$$

## The inequality $\sigma < Q/S$

We turn now to our main objective and prove the inequality $\sigma < Q/S$, $\beta \in (0, \infty)$, fixed $x \in (1, \infty)$. We know that $\sigma < Q/S$ holds for small positive values of *β* since, by (4.2), $\sigma \downarrow 0$ as $\beta \downarrow 0$, and $Q/S > x/(x+2)$ as $\beta \downarrow 0$ by (2.15). Furthermore, $\sigma < Q/S$ as $\beta \uparrow \infty $ since *σ* decreases monotonically toward $\sigma_{\infty }= (x-1)/(x+1) < 1$ as $\beta \uparrow \infty $ by (4.2), whereas $Q/S \uparrow 1$ by (2.14). (The fact that $\sigma_{\infty }< 1$ also follows directly from $\sigma < A < A_{ \infty }< 1$ for $\beta \in (0, \infty)$.) Therefore, if the desired inequality $\sigma < Q/S$ should not hold throughout, it must be violated somewhere in the interval $(0, \infty)$.

Observing definition (4.1) of *σ* and setting $Q/S = z$ for simplicity, the proposed inequality $\sigma < z$ can be transformed into

5.1 $$\begin{aligned}& AB - (1+z)A + z > 0. \end{aligned}$$

Here we set $B = \lambda A$, $\lambda = \lambda (\beta; x) > 1$, $\beta \in (0, \infty)$, $x \in (1, \infty)$. *λ* has a negative *β*-derivative,

$$\lambda ' = -2\lambda (S-T), $$

so that *λ* is strictly monotonically decreasing. By (1.6) and (1.5b),

$$\lambda (1) = \frac{x^{2}}{x^{2} - 1},\qquad \lambda_{\infty }= \lim _{\beta \uparrow \infty } \lambda = \frac{B_{\infty }}{A_{\infty }} = \frac{x^{3} (x+2)}{(x^{2}-1)(x+1)^{2}} > \lambda (1), $$

and, by (1.1) and (1.2), the factors of the infinite product $\lambda = B/A =\kappa^{-1}$ go to $x^{3} (x+2) / (x+1)^{3} (x-1) > 1$ as $\beta \downarrow 0$, i.e., *λ* diverges ↑∞, as $\beta \downarrow 0$.

With $B = \lambda A$, inequality (5.1) takes the form

5.2 $$\begin{aligned}& h(A) = A^{2} - \frac{1+z}{\lambda }A + \frac{z}{\lambda } > 0. \end{aligned}$$

Suppose $h(A)$ as function of *β* were negative somewhere in $(0, \infty)$. Then *h* would have to be zero somewhere in that interval. The roots of the equation $h = 0$ are

5.3 $$\begin{aligned}& A_{1,2} = \frac{1+z}{2\lambda } \pm \frac{1}{\lambda } \bigl[ z^{2} - 2(2\lambda - 1)z + 1 \bigr] ^{1/2}. \end{aligned}$$

Here $(1+z)/2\lambda < 1$ because $1 + z < 2$ and $\lambda > 1$, and the radicant in (5.3)

5.4 $$\begin{aligned}& j (\beta) = z^{2} - 2(2\lambda - 1)z + 1 = (z+1)^{2} - 4\lambda z \end{aligned}$$

is negative as $\beta \downarrow 0$ since $\lambda \uparrow \infty $, and, as $\beta \uparrow \infty $, by (1.5b) and with $z \uparrow 1$,

$$j_{\infty }= -4 \frac{2x+1}{(x-1)(x+1)^{3}} < 0. $$

Thus, for small and large values of *β*, $A_{1,2}$ would be nonreal.

Suppose *j*, given in (5.4), were positive somewhere in $(0, \infty)$. Then it must be zero somewhere in that interval. The equation $j=0$ has the two roots

$$\begin{aligned}& z_{1} = (2\lambda - 1) - 2 \sqrt{\lambda ( \lambda -1)}, \quad 0 < z < 1, \\& z_{2} = (2\lambda - 1) + 2 \sqrt{\lambda (\lambda - 1)}. \end{aligned}$$

The second one is greater than unity since $\lambda > 1$. It is outside the range of $z = Q/S < 1$. Then, using the root $z_{1}$ for *z* in (5.3) and squaring the equality to get rid of the square root, we arrive at the equality $\lambda A^{2} - 2\lambda A + 1 = 0$, or, if we replace *λA* by *B* and *AB* by *C*, at the equality

$$C - 2B + 1 = 0, $$

which we have encountered before in (4.11). We know that it holds if and only if $\beta = 1$. But for $\beta = 1$,

$$\sigma (1) = \frac{x-1}{x+1},\qquad \frac{Q(1)}{S(1)} = \frac{3x +1}{3(x+1)} > \sigma (1). $$

This contradiction shows that $h(A)$ cannot be negative or zero on the interval $0 < \beta < \infty $. Thus, (5.2) holds, and $\sigma < Q/S$ for $\beta \in (0, \infty)$, fixed $x \in (1, \infty)$.

## Declarations

### Results and discussions

Inequalities have been proved which involve various combinations of psi- and hypergeometric functions. They add to the wealth of knowledge in the theory of these special function classes of higher analysis.

### Conclusions

The main inequality of this paper guarantees uniqueness of the hyper-gamma parameter estimation and its application. Usefulness of this approach has been demonstrated in [6].

### Methods/experimental

The aim of the study is to prove an inequality made up of functions of higher mathematical analysis. This inequality guarantees monotonicity of the first moment equation function of the four-parameter hyper-gamma probability density estimation problem. Monotonicity guarantees uniqueness of the numerical solution process. Standard analytical methods of higher analysis have been employed to accomplish the proof.
